# Science for Pandemic Preparedness: A Precautionary Framework

**DOI:** 10.1111/jep.70309

**Published:** 2025-11-02

**Authors:** David Kriebel, Rok Ho Kim, Trisha Greenhalgh

**Affiliations:** ^1^ Lowell Center for Sustainable Production University of Massachusetts Lowell Lowell Massachusetts USA; ^2^ Institute for Planetary Health Yonsei University Seodaemun‐gu Republic of Korea; ^3^ Nuffield Department of Primary Care Health Sciences University of Oxford Oxford UK

**Keywords:** COVID‐19, decision making, health policy, pandemic preparedness, pragmatism, precautionary principle

## Abstract

In the early weeks of the pandemic year 2020, health agencies were slow to warn of the potential for a global health emergency while scientists waited for ‘sufficient’ evidence of human‐to‐human transmission of the new coronavirus. There were further delays and confusion in issuing guidance because of uncertainty about the most important routes of exposure. A 2021 international expert panel review of COVID‐19 pandemic response concluded: ‘The bias of the current system of pandemic alert is towards inaction—steps may only be taken if the weight of evidence requires them. This bias should be reversed—precautionary action should be taken on a presumptive basis, unless evidence shows it is not necessary’. To implement this recommendation, health agencies should incorporate the concept of precaution into their methods for rapidly and transparently reaching policy decisions. In this article, we summarise the historical development of precautionary policy frameworks, and present 4 key components. First, take action if potential risks are large, even when the science is uncertain. This corresponds to the common sense idea of ‘better safe than sorry’. Second, consider the burden of proof required before choosing among alternative actions. Third, ensure that the full range of potential solutions is considered by broadly defining the problem and consulting all relevant scientific fields. Finally, increase public participation so that all relevant perspectives are included, and the chances of public buy‐in are increased. Drawing lessons from past applications and missed opportunities, we propose steps that health agencies could take to incorporate precaution into a pragmatic approach to using science for pandemic preparedness and response. The risk of another pandemic from the highly pathogenic avian influenza A (H5N1) virus underscores the urgency of this new approach.

## 1 Introduction

The World Health Organisation (WHO) declared the end of the COVID‐19 public health emergency in May 2023, but the risk of future pandemics remains high [1]. The highly pathogenic avian influenza A (H5N1) virus provides just one current example. An outbreak in the US began in March 2024 when the virus was detected in several herds of dairy cows. The disease has also spread through flocks of domestic and wild birds, domestic cats, and as of July 2025, a small but growing number of humans. Despite these worrisome trends, the US Centres for Disease Control and Prevention considered the health risk to the general public from H5N1 to be low [2]. There is however, concern that continued circulation in domestic animal populations could lead, following an uncertain number of random mutations, to a new strain with efficient human‐to‐human transmission, at which point it could spread rapidly and with very serious consequences [3].

This is much the same situation that the world faced in the winter of 2019−2020 with SARS‐CoV‐2, and before that in 2003−2004 with SARS‐CoV‐1. In both cases, public health authorities were slow in responding adequately because of uncertainties about the risk of transmission. And in both cases, subsequent independent expert reviews of the public health responses found the same mistake: waiting for ‘sufficient’ scientific evidence instead of taking a precautionary approach [4, 5, 6].

Critiques of the hesitant responses of health authorities to both SARS outbreaks invoked the precautionary principle (PP) as a policy that might have resulted in quicker preventive actions. The PP calls for acting in the face of uncertainty when there are potentially large or urgent hazards, and it has been called for (but often ignored) in response to ecologic and health threats from chemical contaminants. Precaution is often seen as ‘unscientific’ and contrary to the accepted wisdom that policymakers should simply ‘follow the science’.

In this article, we present the PP and how it might improve pandemic preparedness. We argue that rather than unscientific, the PP is a pragmatic approach to managing uncertainty; entirely consistent with policymaking based on the best available evidence.

There have been numerous evaluations and critiques of the public health responses to the COVID‐19 pandemic. One of the most thorough‐going and comprehensive was conducted for WHO. The World Health Assembly in May of 2020 called on the WHO Director‐General to conduct an independent review to identify lessons learned from the actions of WHO and Member States, and recommend steps to improve future pandemic preparedness and prevention [7]. One year later, in May 2021, the Independent Panel for Pandemic Preparedness and Response (IPPPR), co‐chaired by Right Honourable Helen Clark and Her Excellency Ellen Johnson Sirleaf, published a comprehensive report which identified critical lessons and urged comprehensive reforms at many levels of international public health preparedness [5].

The Independent Panel's recommendations included:

- International leadership and coordination, including a Pandemic Treaty, and a new Global Health Threats Council;
- Rapid and substantial increases in investment in preparedness;
- A dramatically expanded global platform for producing and disseminating vaccines, diagnostics and other essential supplies; and
- Improved surveillance and rapid alert systems.

The IPPPR found there was a need for a new international system for surveillance and alert, after finding limitations in the COVID‐19 pandemic response:

The bias of the current system of pandemic alert is towards inaction—steps may only be taken if the weight of evidence requires them. This bias should be reversed—precautionary action should be taken on a presumptive basis, unless evidence shows it is not necessary’. The report went on: ‘While WHO advised of the possibility of human‐to‐human transmission in the period until it was confirmed, and recommended measures that health workers should take to prevent infection, the Panel's view is that it could also have told countries that they should take the precaution of assuming that human‐to‐human transmission was occurring. Given what is known about respiratory infections, there is a case for applying the precautionary principle and assuming that in any outbreak caused by a new pathogen of this type, sustained human‐to‐human transmission will occur unless the evidence specifically indicates otherwise.

## 2 What Is the PP?

‘Better safe than sorry’ has probably always been a common‐sense heuristic throughout human history. The PP per se was formalised in the 20th century out of concerns for protecting the environment from pollution [8]. An early and influential assertion of the importance of the PP in international policy was Principle 15 of the United Nations Rio Declaration on Environment and Development of 1992: ‘To protect the environment, the precautionary approach shall be widely applied by States according to their capabilities. Where there are threats of serious or irreversible damage, lack of full scientific certainty shall not be used as a reason for postponing cost‐effective measures to prevent environmental degradation’ [9]. Since its original application as a guide to protecting the environment, the PP has been defined and applied in many other settings [10, 11, 12, 13, 14, 15].

### 2.1 Four Central Components of the PP

The 1998 Wingspread Statement on the PP provided one of the clearest definitions: ‘When an activity raises threats of harm to human health or the environment, precautionary measures should be taken even if some cause and effect relationships are not fully established scientifically’ [16]. The statement went on to list four central components of the PP:

1. Taking preventive action in the face of uncertainty;
2. Shifting the burden of proof to the proponents of an activity;
3. Exploring a wide range of alternatives to possibly harmful actions; and
4. Increasing public participation in decision‐making.

#### 2.1.1 Acting Under Uncertainty

This seems to be broadly understood, and what most people, including the IPPPR had in mind when speaking about precaution. But the PP requires an expansive understanding of what is meant by ‘uncertainty’. Standard approaches to synthesising and evaluating evidence (in evidence‐based medicine, e.g.,) may miss deeper, more systemic sources of uncertainty including ambiguities in how the problem and solutions are defined and measured, as well as ignorance about essential aspects of the problem and potential solutions.

#### 2.1.2 Shifting the Burden of Proof

In the context in which the PP was first defined, shifting the burden of proof meant expecting those who profit from some new technology to demonstrate absence of harm, rather than the typical situation in which those who may be harmed are required to ‘prove’ that the technology is to blame. The concept has a deeper meaning though, that is relevant across a wider range of health issues. The ‘burden of proof’ in this wider arena means the weight of evidence needed to recommend one action over another. This burden depends on context. Decision making in clinical practice tends to require a greater weight of evidence to recommend an intervention (a treatment or drug typically) than to do nothing; a logic that follows directly from the physician's obligation to ‘first do no harm’. But in many public health decisions, there may not be such a compelling case for requiring more evidence to take an action than not to act, as the example of masking during the COVID‐19 pandemic illustrates.

One of the first, urgent questions from the first months of the pandemic was the efficacy of masking in public spaces to prevent disease spread. Critics of masking argued that there was little evidence that masks were effective, which was used to argue that they therefore *should not* be worn. Instead, perhaps the burden should have been on those who did not want masking to demonstrate their *ineffectiveness*, and lacking such evidence, the PP would lead to the recommendation to wear masks [17, 18, 19].

One way to think about the burden of proof is by attention to the risks of ‘false positive’ and ‘false negative’ mistakes, or what statisticians call Type I and Type II errors. Type I errors occur when one falsely concludes that a finding is ‘real’ or ‘significant’ when in truth it is not. So in the case of masks, declaring that they work—if in truth they do not, while a Type II errors would mean deciding that masks do not work when in truth they do. Health science often places more emphasis on minimising Type I than Type II errors, but taking a precautionary approach, one might choose to reduce the risk of a Type II error—emphasising safety over a risk of falsely declaring something is a hazard (better safe than sorry) [20].

#### 2.1.3 Ensuring Broad Problem and Solution Framing

The search for alternatives to a potentially hazardous product or action is another important common theme of the PP. Methods for synthesising evidence for policy making (evidence to decision frameworks) that, intentionally or not, encourage narrowing of data evaluations to simple side‐by‐side comparisons of limited options risk missing important alternatives. Different scientific disciplines tend to develop their own methods of problem framing and problem solving, and languages to describe these. Causal inference, perhaps the most fundamental task for science guiding policy, is approached very differently by different disciplines [21]. These different science cultures can make it very difficult to work across disciplinary boundaries, and prejudices can develop in which one's own methods are thought to be superior. The PP calls for transdisciplinary consultation with experts collectively possessing the widest possible range of expertise. This can increase the chances that all relevant perspectives, tools and wisdom contribute to recommendations.

#### 2.1.4 Increasing Public Participation in Decision‐Making

This fourth component of the PP rests on several assumptions. Non‐experts have not been trained to think from narrow disciplinary frames of reference, and may make connections that scientists may miss, and contribute social and political values bearing on the risks and benefits being considered and how these might differ across populations [22]. Also, in democratic societies, transparency and public trust must be maintained if policies are to be effective, and so consultation with broad representation of society may increase public support.

### 2.2 Criticisms of PP

The PP has been criticised in several substantive ways. It has been dismissed as too abstract, because it does not specify how much precaution should be taken in a given situation, nor does it provide strong guidance on when precaution should be invoked [20]. These apparent weaknesses have led some to dismiss the PP as unscientific [23]. Critics point out that all actions entail risks, and applying precaution means focusing on some potential risks to the exclusion of others, rendering the PP fundamentally incoherent and liable to lead to unintended consequences and potentially even greater risks [23]. For example, it has been suggested that applying the PP might lead to a ban on the pesticide DDT, which not only has chronic human health effects, but is environmentally persistent, bioaccumulates and has widespread impacts on ecosystem health [24]. DDT is an inexpensive and often effective way to control malaria by killing the vector mosquito. Thus, if DDT were banned, malaria might increase, substantially offsetting any health and environmental benefits from the DDT ban. There are several flaws in this critique of precaution. The PP would not ‘automatically’ lead to a ban, but would instead lead to a broad review of the full range of health and environmental benefits and risks of its use. There would be a search for alternative ways of reducing malaria, and the benefits and risks of these alternatives would also be evaluated with the best available evidence. The apparent ‘false positive’ error arises from framing the problem too narrowly as having only two choices: spray DDT or allow malaria to spread. One way to ensure the widest possible problem and solution framing would be to include members of the affected communities in the assessment—a key component of the PP.

## 3 Precaution Is Pragmatic

The criticism that precaution is unscientific may stem from misunderstanding of the kind of science that is appropriate for informing health policy [25, 26]. Current practices in health science research place a high value on objectivity, repeatability and reductionism. These attributes are important elements of scientific research, but are often insufficient for confronting urgent problems with highly uncertain and limited evidence. Instead of such currently favoured approaches as evidence‐based medicine, the philosopher of science Maya Goldenberg proposes that the 150‐year tradition of the philosophy of pragmatism is more appropriate when collecting and interpreting evidence bearing on an urgent public health question like the mode of transmission of the SARS‐CoV‐2 virus [27].

Pragmatism holds that the value of science derives from its usefulness in the real world, not from the creation of some pure or abstract truth [26, 27]. All scientists recognise that scientific truths are rarely absolute and evolve over time as knowledge grows. But when problems are urgent and evidence uncertain, beliefs about what is true are often contested. In such circumstances, often the best we can do is to use evidence from real‐world experience to decide between competing beliefs, while keeping those decisions open to revision as new evidence comes in. All branches of human knowledge, not just ‘hard’ sciences, can be valuable for understanding the world and guiding action. Before deciding how best to test a specific hypothesis, pragmatists want to know what will be done with the result; what are the implications or consequences of rejecting or failing to reject the hypothesis? These implications should have direct bearing on how the study is designed. The PP fits very well into the framework of pragmatic science [26].

## 4 Precaution and the COVID‐19 Pandemic

### 4.1 SARS—Lessons Not Learned

The COVID‐19 epidemic was not the first time that health authorities failed to take precautionary action against a SARS virus. The Ontario SARS Commission, reviewing the successes and failures of the provincial health system in its response to the epidemic of a novel viral illness in 2003−2004, made remarkably similar recommendations [4]. The respiratory disease that came to be known as SARS was first reported in Guangdong Province, China in late 2002 and spread rapidly to 29 countries, territories and areas, causing an estimated 774 deaths over 6 months. The SARS outbreak in Ontario Canada killed 44, and severely sickened hundreds. Many of the deaths were among health care personnel treating infected patients. The Ontario SARS Commission, led by the late Justice Archie Campbell (1942−2007), found that there was confusion and controversy around the mode of transmission of the virus, and the resulting level of respiratory protection that was needed, particularly for health care workers.

The Ontario report found that there were costly and avoidable delays in determining what equipment and training were needed for health care personnel to protect themselves from a novel viral infection [4]. The report concluded: ‘Perhaps the most important lesson of SARS is the importance of the precautionary principle. SARS demonstrated over and over the importance of the principle that we cannot wait for scientific certainty before we take reasonable steps to reduce risk. This principle should be adopted as a guiding principle throughout Ontario's health, public health and worker safety systems…There is no longer any excuse for governments and hospitals to be caught off guard, no longer any excuse for health workers not to have available the maximum reasonable level of protection through appropriate equipment and training, and no longer any excuse for patients and visitors not to be protected by effective infection control practices’.

### 4.2 Transmission of SARS‐CoV‐2

The IPPPR found that WHO was slow to warn of the potential for a global epidemic while scientists waited for sufficient evidence of human‐to‐human transmission of the new coronavirus [5]. Once this was established, there were further delays in making clear recommendations because of uncertainty about the most important routes of exposure. The Lancet Commission on lessons for the future from the COVID‐19 pandemic pointed to the failure of international authorities to acknowledge the importance of the airborne route of exposure to SARS‐CoV‐2 until many months into the pandemic [6].

These delays represent missed opportunities for precautionary action. In the first instance, the IPPPR specifically pointed out that the burden of proof for the question of human‐to‐human transmission should have been shifted, leading to WHO telling member states that they should assume there was human‐to‐human transmission until evidence showed clearly that this was false. Instead, they waited for sufficient evidence of transmission before issuing their warning. On the question of the important routes of exposure, several investigators have noted that evidence of airborne transmission of SARS‐CoV‐2 in indoor spaces with poor ventilation had been identified very early in the pandemic, but was largely overlooked because of differences between scientific disciplines over how to evaluate evidence [28, 29, 30]. A precautionary approach might have ensured that the full range of relevant scientific disciplines was included in the deliberations—not only clinical specialties like infectious disease but also aerosol science, environmental epidemiology and occupational hygiene [31].

## 5 Putting Precaution Into Practice: What Needs to be Done

Public health agencies preparing for the next pandemic should incorporate the PP into their operational guidance. Precaution should be viewed as both a fundamental value like health equity, as well as a logically‐derived operational principle.

### 5.1 An Institutional Commitment to Precaution

During a crisis, when there is an urgent need for scientific guidance, invoking the PP would underline an agency's commitment not only to the best science but also to maintaining trust, transparency and humility. Developing the scientific basis for responding to emergencies will often require methods different in scope and content, and not simply in speed, from current procedures. Initiating these substantive changes in methods and approach can best be accomplished by setting the PP alongside existing commitments such as to using the best available evidence and to respecting health equity and human rights. Precaution does not contradict a best available evidence approach, and in times of need for urgent action, precaution would actually strengthen the commitment to the best science. Also, the values of health equity and of precaution are closely linked. The COVID‐19 pandemic has once again demonstrated that when there are delays in global public health response, it is usually the poor and vulnerable who suffer most.

Building an institutional commitment to precaution might involve these components:

- Leadership commitment to the PP, as well as support of staff for their ongoing engagement;
- Training of staff to recognise the need and opportunity to invoke the PP throughout all phases of the development of policy recommendations;
- Explicit incorporation of precautionary considerations into standard methods for developing and communicating public health guidance; and
- Establishing accountability mechanisms and monitoring effectiveness of initiatives for integrating precaution into evidence synthesis and evaluation.

### 5.2 Incorporating the PP Into Standard Evidence to Decision Protocols

Public health agencies should review existing methods to identify opportunities for introducing precautionary considerations into their policy guidance. This review might be organised around the four key themes of the PP noted above (Table 1).

**Table 1:** The precautionary principle in action. A checklist for public health agencies developing urgent policy recommendations.

Key considerations	Checklist items
Acting under uncertainty	How can the evidence to decision framework be modified to ensure that a wider field of types of evidence is admissible? Is the evidence to decision framework flexible enough to identify when evidence is limited in an absolute sense, but adequate to recommend low‐risk interventions? How can the evidence to decision framework be modified to avoid preferences for certain types of scientific training and tools to the detriment of others?
Considering the burden of proof	When comparing alternative interventions, is there a greater burden of proof in choosing one option over another? Is this appropriate? Are the methods of evidence synthesis setting Type I and Type II error rates in a way that makes it harder/easier to reach a precautionary recommendation?
Ensuring broad problem and solution framing	How might the range of alternative interventions that are evaluated be expanded? How might the range of potential beneficial and negative consequences of interventions be expanded? How can the evidence to decision framework be modified to ensure that the full range of relevant scientific disciplines is included? Are the methods for evidence synthesis inadvertently leading to the exclusion of certain types of evidence that, in an emergency, should be included?
Increasing public participation	Are non‐experts being appropriately included in evidence evaluation and decision making? Are disproportionately affected communities being appropriately represented?

#### 5.2.1 Acting Under Uncertainty

Systems to enable precautionary action require attention to all sources of uncertainty. Beyond the all‐too‐common problem of few available studies bearing on an urgent question, there are often also ambiguities in how the problem and solutions are defined and measured, as well as complete ignorance about what are later revealed to be critical aspects of the problem. Because of these knowledge gaps, the likelihood that actions taken under uncertainty will ultimately be the right ones will be increased by getting input from the widest range of expertise.

#### 5.2.2 Shifting the Burden of Proof

Because of its roots in clinical practice, evidence based medicine tends to require a greater weight of evidence to recommend an intervention (a treatment or drug typically) than to do nothing [20]. But in many public health decisions, a low‐cost or low‐risk intervention may be prudent, even if benefits are uncertain. Small individual effects can result in substantial impacts when implemented across a population. At a minimum, attention to the PP means an explicit recognition of how much evidence is ‘enough’, and whether the amount of evidence needed for recommending a precautionary action is appropriately weighted given the likely consequences.

#### 5.2.3 Ensuring Broad Problem and Solution Framing

Evidence synthesis and evaluation methods that, intentionally or not, encourage narrowing of data evaluations to simple side‐by‐side comparisons of limited options risk missing important alternatives. The PP calls for transdisciplinary consultation with experts possessing the widest possible range of expertise. This can increase the chances that all relevant perspectives, tools and wisdom contribute to recommendations.

#### 5.2.4 Increasing Public Participation

Non‐experts see the world differently and may question assumptions built into standard scientific training and methods. There are many good models of formal processes for gathering and incorporating non‐expert knowledge into technical guidance, including community or citizens' juries, town hall meetings and community forums [32].

## 6 Conclusions

In a 2023 Health Policy paper in this journal, Torreele and colleagues laid out a plan for transforming the global health architecture to focus on public health need generally, and improved pandemic preparedness and response specifically [33]. While they did not discuss precaution explicitly, they argue for a pragmatic approach to science for policy into which the PP fits logically. During a crisis, when there is an urgent need for scientific guidance, invoking the PP could support not only the best science but also help public health authorities maintain public trust, transparency and humility. The PP can help decision‐making under uncertainty be more agile and responsive, while remaining committed to using the best available evidence.

## Author Contributions

D.K. conceptualised the commentary and wrote the original draft with significant input from the other two authors. All three authors reviewed and edited the manuscript.

## Conflicts of Interest

T.G. is a member of Independent SAGE and is a co‐investigator on a grant from UK Research and Innovation to study evidence synthesis and epistemic justice using masks as a worked example. The other authors declare no conflicts of interest.

## Data Availability

Data sharing not applicable to this article as no data sets were generated or analysed during the current study.
